# MicroRNA Silencing by DNA Methylation in Human Cancer: a Literature Analysis

**DOI:** 10.3390/ncrna1010044

**Published:** 2015-04-20

**Authors:** Ziga Strmsek, Tanja Kunej

**Affiliations:** Department of Animal Science, Biotechnical Faculty, University of Ljubljana, Groblje 3, Domzale 1230, Slovenia; E-Mail: zstrmsek@gmail.com

**Keywords:** cancer, DNA methylation, epigenetics, microRNA (miRNA), oncomiRs

## Abstract

MicroRNAs (miRNAs) are small non-coding RNAs that post-transcriptionally regulate the expression of target mRNAs. MicroRNA genes themselves are regulated through epigenetic mechanisms, such as histone modifications and/or DNA methylation of CpG islands. Aberrant CpG island methylation patterns are frequently associated with cancer and thus researching DNA methylation of miRNA genes is a topic of increased research interest. Large quantities of available data from various studies are fragmented and incomplete; therefore, integration was performed. Data from 150 articles revealed 180 miRNA genes shown to be regulated via DNA methylation in 36 cancer types. From the total of 2588 known mature miRNA 6.9% (180/2588) miRNAs have been shown to be epigenetically regulated by DNA methylation. 45.5% (82/180) of miRNA genes were shown to be methylated in at least two cancer types among them hsa-miR-34b, hsa-miR-34c and hsa-miR-34a were found to be silenced in 24, 21 and 17 cancer types, respectively. The other 54.4% (98/180) miRNA genes regulated by DNA methylation were found to be specific for a certain type of cancer and therefore represent specific biomarker potential. Because specific miRNAs have diagnostic, prognostic and therapeutic potential, the systematically review of the field offers an overview of the field and facilitates experiment planning, generation of more targeted hypotheses and more efficient biomarker and target development.

## 1. Introduction

MicroRNAs (miRNAs) are small non-coding RNAs (19–25 nucleotides in length) that post-transcriptionally regulate the expression of target mRNAs and it is estimated that they regulate approximately two thirds of human genes [1]. MicroRNA genes can be regulated through epigenetic silencing with histone modification and/or DNA methylation of CpG islands that encompass or lie adjacent to miRNA genes. Moreover, it appears that the frequency of DNA methylation of miRNA genes and consequently the frequency of epigenetic regulation are at about one order of magnitude higher than that of the protein encoding genes [2,3].

MicroRNAs that are associated with cancer are called oncomiRs and they have been linked with cancer in early stages of miRNA research because they: (1) are involved in regulation of cell proliferation and apoptosis [4,5]; (2) were found to be deregulated in malignant tumors and tumor cell lines in comparison with normal tissues [6,7,8]; and (3) are located on breaking-prone and cancer-associated genomic regions [9]. MicroRNAs can represent two opposing roles, either behaving as oncogenes or tumor suppressors depending on the tissue type and presence of specific targets [10]. Additionally, miR-34 gene family has also been identified to be silenced by DNA methylation in the highest number of cancer types and thus indicated high therapeutic potential [11]. Moreover, miRNA based drug MRX34 has already entered phase I clinical studies [12].

Since the first publication in 2006 [13] which proved an epigenetic regulation of *hsa-miR-127* gene, a lot of studies have reported that miRNAs are epigenetically regulated in different cancer types, tissues and cell lines. Cancer cells and miRNA genes are subjected to aberrant DNA methylation of adjacent CpG islands. DNA methylation patterns of miRNA genes are linked with cancer development, growth, metastasis, *etc.* Consequently, DNA methylation of miRNA genes in cancer has been a topic of increased research interest. Due to sheer quantity of available information an integration of available data regarding miRNA regulated by DNA methylation is needed followed by continuous systematic reviews, which would integrate incomplete and fragmented results from various studies. Thus, we performed a systematic literature integration of all available data of miRNA genes regulated by DNA methylation in cancer and upgraded our previous study [2]. We integrated the data of 150 publications in order to identify epigenetically regulated miRNAs, specific for cancer types and common to different cancer types. We organized oncomiRs regulated by DNA methylation according to association with different cancer types and added additional available data of miRNA genes regulated by DNA methylation in cancer including miRNA-target interactions and tissue specific DNA methylation of miRNA genes. Furthermore, we proposed miRNA genes that possess cancer type biomarker potential.

## 2. Results

**Figure 1:**
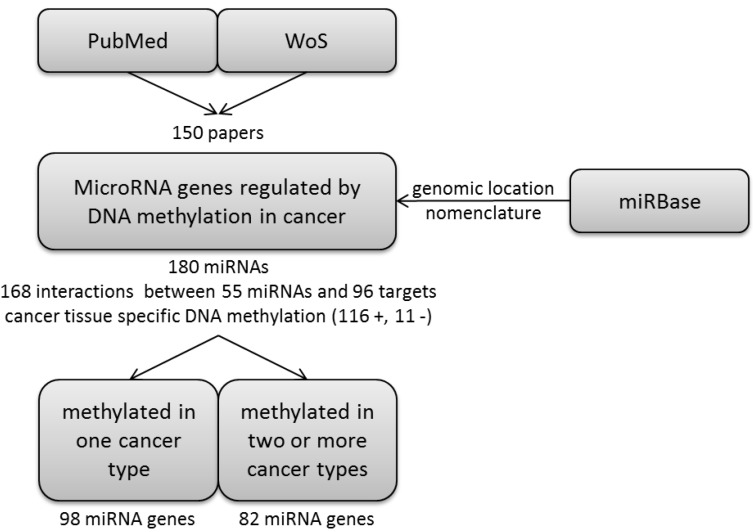
Workflow of the study and main results. We reviewed scientific articles and extracted information regarding microRNA genes regulated by DNA methylation in cancer. The goal of the following analysis was to differentiate between miRNA genes whose regulation by DNA methylation is common to two or more cancer types and to those that are associated with one cancer type.

We performed a systematic review of literature and organized the extracted data of epigenetically regulated miRNA genes in cancer. The workflow and main results are presented in Figure 1.

### 2.1. Systematic Review of Literature of miRNA Genes Regulated by DNA Methylation

Based on data integration of 150 articles (List S1), 180 oncomiR genes have been proven to be regulated by DNA methylation in 36 cancer types. The number of miRNA genes regulated by DNA methylation varied greatly between cancer types; from one in multiple myeloma to 72 in gastric cancer. From the total of 2588 known mature miRNA 6.9% (180/2588) miRNAs have been shown to be epigenetically regulated by DNA methylation. 45.5% (82/180) of miRNA genes were shown to be methylated in at least two cancer types. The other 54.4% (98/180) of miRNA genes were found to be regulated DNA methylation in only one type of cancer and thus possess cancer type specific biomarker potential. Moreover, DNA methylation status of miRNA genes against adjacent tissue was also investigated in 61 out of 150 studies; and our study revealed 116 associations of miRNA genes and cancer types where DNA methylation status differed between cancerous and adjacent tissue and 11 where it was the same. Integration of data also revealed 168 interactions between 55 miRNAs and 96 target mRNAs.

### 2.2 MicroRNA Genes Methylated in More Than Two Cancer Types

**Figure 2:**
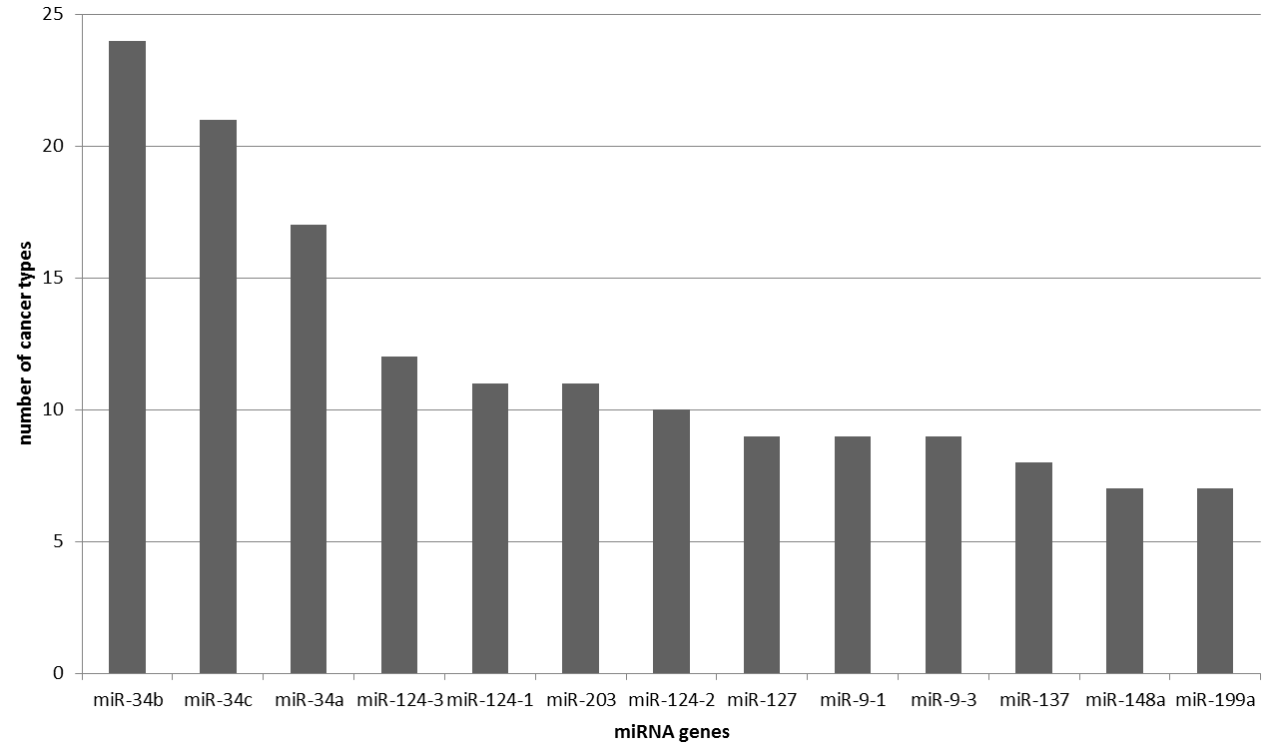
MicroRNA genes shown to be silenced by DNA methylation in more than seven cancer types.

Eighty-two out of 180 miRNA genes regulated by DNA methylation were found to be epigenetically regulated in two or more cancer types. The analysis showed that 13 miRNA genes have been common to seven or more different cancer types (Figure 2). MicroRNA genes miR-34b, miR-34c and miR-34a were found to be associated with the highest number of cancer types; with 24, 21 and 17 cancer types, respectively. Gene *hsa-miR-127,* commonly used as a positive control, has been associated with nine cancer types.

### 2.3. MicroRNA Genes Methylated in One Cancer Type

**Figure 3:**
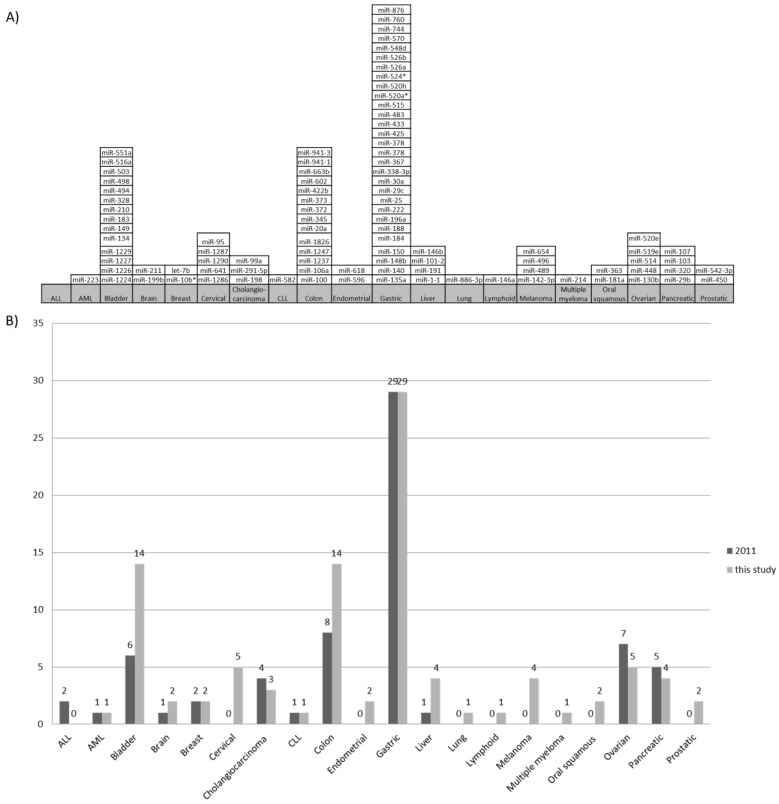
A graph showing miRNAs that are associated with one cancer type. (**A**) A list of miRNA genes from the 2015 update study that have been shown to be regulated by DNA methylation in one cancer type and thus possess cancer type specific biomarker potential, (**B**) Comparison of epigenetically regulated cancer-specific miRNA genes by cancer type between study [2] and this study, based on 45 and 150 papers, respectively. ALL - acute lymphoblastic leukemia, AML - acute myeloid leukemia, CLL - chronic lymphocytic leukemia

Out of 180 oncomiR genes regulated by DNA methylation in 36 cancer types, based on current data 98 were found to be associated with only one cancer type and thus possess potential to be cancer-specific (Figure 3A). MicroRNA genes, associated with only one cancer type were revealed in 19 out of 36 cancer types. The highest number of studies was performed on gastric and colon cancer. Out of those 98 miRNA genes, 72 were shown to be regulated by DNA methylation in gastric cancer; 29 out of 72 were found to be specific only for gastric cancer. Additionally, from 54 miRNA genes regulated by DNA methylation associated with colon cancer, 14 were found to be regulated by DNA methylation only in colon cancer. DNA methylation pattern against adjacent tissue was tested in three out of 14 miRNA genes. Two out of those (miR-345 and miR-372) were proven to have specific DNA methylation status against adjacent tissue. Opposing results have been reported for miR-373; as DNA methylation status in the adjacent tissue has been reported to be the same by Lujambio *et al.* [14], and specific for cancer tissue in the study by Tanaka *et al*., [15]. For the remaining 11 out of 14 genes, associated to colon cancer (miR-100, -106a, -1237, -1247, -1826, -20a, -422b, -602, -663b, 941-1 and -941-3) DNA methylation pattern against adjacent tissue was not tested.

MicroRNA genes associated with only one cancer type and different DNA methylation status from adjacent tissue hold specific cancer type biomarker potential. Moreover, data regarding development of biomarkers will change with adding more data from upcoming publications, especially for cancer types with only single study present at this time (e.g., esophageal cancer). However, the highest number of studies was performed on gastric and colon cancer and this data currently provides the most reliable source of information for biomarker development.

## 3. Discussion

Out of all associations of miRNA genes and cancer types included in presented study about half of miRNA genes (98/180) were associated with only one cancer type while the rest were associated with two or more cancer types. MicroRNA gene associated with the highest number of cancer types was miR-34b, followed by miR-34c and miR-34a. Therefore gene family miR-34 represents top three miRNA genes regulated by DNA methylation in different cancer types.

Our systematic review revealed that there has been some progress of the field since the last integrative study [2]. This study integrates data from additional articles (45 in 2011 and 150 in this study) and it also includes higher number of miRNA genes found to be regulated by DNA methylation (122 genes in 2011 and 180 genes in this study). An update of previous study also resulted in changes of miRNA genes that show cancer-specific DNA methylation patterns (Figure 3B). For some cancer types there was no change; both cancer type and miRNA gene regulated by DNA methylation remained, while for others there was change in number of miRNA genes that have cancer-specific DNA methylation or miRNA genes. For example, our previous study [2] revealed six miRNA genes shown to be methylated only in bladder cancer (mir-134, mir-183, mir-192, mir-429, mir-494, mir-498) while this study reports 14 miRNA genes that have bladder cancer-specific DNA methylation (miR-1224, miR-1226, miR-1227, miR-1229, miR-134, miR-149, miR-183, miR-210, miR-328, miR-494, miR-498, miR-503, miR-516a, and miR-551a), four of those (miR-134, miR-183, miR-494 and miR-498) being the same as reported in [2], while two (mir-192 and mir-429) proved to no longer have bladder cancer-specific DNA methylation. The remaining ten out of 14 miRNA genes (miR-1224, miR-1226, miR-1227, miR-1229, miR-149, miR-210, miR-328, miR-503, miR-516a and miR-551a) have been published after previous study and are thus a result of updated database. As miRNA based drugs (MRX-34) are already well into clinical trials with more therapeutics following it is of upper most importance to understand the mechanisms and pathways involved. As miRNA hold both biomarker and therapeutic potential [16,17], projects like one presented, offer insights into complex epigenetic regulatory networks. In this study we have integrated laboratory proven data that indicates that 98 out of 180 have specific biomarker potential as their DNA methylation status was regulated by DNA methylation in only one cancer type. These miRNA need to be further researched in the future as it could still be proven that their DNA methylation status is not cancer type specific. Furthermore, future research could reveal that DNA methylation status of some miRNA genes are also linked with other conditions. Moreover, because of miRNA’s nature most miRNAs that have biomarker potential also poses therapeutic potential thus potentially eliminating the need for sophisticated target delivery systems that limit development of cancer therapeutics at the moment. The remaining 82 out of 180 miRNA genes that have been proven to be regulated by DNA methylation in two or more cancer types have general cancer biomarker and therapeutic potential but follow-up studies should be performed to determine DNA methylation status of miRNA genes against adjacent tissue.

## 4. Future Perspectives

Our systematic review and findings of a recent critical appraisal by Lehman *et al.* [18] indicated that the planning of research experiments of epigenetically regulated oncomiR genes is still not optimized. The majority of studies published recently tested DNA methylation of miRNA genes as an additional experiment and not as main aim of the study. Researchers should focus on miRNA genes that have already been identified to be regulated by DNA methylation and retest their biomarker potential. DNA methylation status of miRNA genes should be determined for several cancer types and their adjacent tissue. It is likely that epigenetic regulation of some miRNA genes will also be revealed in other cancer types. Moreover, it would be necessary to test if methylation status of miRNA genes is cancer specific or also present in other diseases. Extending data integration of DNA methylation status across several diseases would further facilitate development of the field towards clinical application. Furthermore, identification of function of miRNA genes regulated by DNA methylation in cancer should also become an additional goal of future studies. It is also worth noting that DNA methylation is not the only factor that should be taken into account when researching expression of miRNA genes – histone modifications, miRNA cross talk, overlapping genes (host genes), genetic variability, *etc*. also play an important role in biology of miRNA genes and should thus be taken into account when researching expression of miRNA genes in cancer. Moreover, the collected data related with miRNA-target interactions will enable to reveal miRNA regulatory networks.

At the moment it is not yet possible to establish gold standard for research of miRNA genes regulated by DNA methylation but this study was performed to facilitate development and offer integrative overview of the field. In order to achieve that the following propositions were made: (1) in future researchers should retest methylation status if miRNA genes, that have already been proven to be regulated by DNA methylation, but don’t have available information regarding DNA methylation status against adjusted tissue (e.g., in colon cancer miR-100, -106a, -1237, -1247, -1826, -20a, -422b, -602, -663b, 941-1 and -941-3); (2) future studies should revalidate the DNA methylation status of cancer-specific miRNA genes that based on the current data have been shown to be methylated by only one study. The proposed decision scheme was previously introduced in Strmsek *et al.* [11] with the goal of offering researchers a baseline for planning research experiments and thus facilitate development of the field and enable higher efficacy. We also ask researchers to send us potentially overlooked publications related to this topic, so they can be included in the next update study.

## 5. Material and Methods

We reviewed the literature published from 2006 to 9/2014 searching for the relevant publications through PubMed (http://www.ncbi.nlm.nih.gov) and WoS (https://webofknowledge.com) using key phrases: non-coding RNA, microRNA, oncomiR, epigenetic gene regulation, DNA methylation, CpG island, cancer, oncogene. Location of miRNA genes was obtained from microRNA Registry (miRBase), release 21 June 2014 [19]. The miRNA nomenclature that varied between different studies was made uniform according to the miRBase and gene nomenclature was made uniform according to HUGO Gene Nomenclature Committee.

## Supplementary Files

Supplementary File 1
