# Supplementary material for: MicroRNA Silencing by DNA Methylation in Human Cancer: a Literature Analysis
Source: Noncoding RNA. 2015 Apr 20;1(1):44–52. doi: 10.3390/ncrna1010044 (PMC5932538; doi:10.3390/ncrna1010044)
Supplement: Supplementary File 1 [file ncrna-01-00044-s001.docx]

**Supplementary Materials**

MicroRNA Silencing by DNA Methylation in Human Cancer: a Literature Analysis

Ziga Strmsek and Tanja Kunej *

**List 1.** Articles analyzed in our article

1. Agirre, X., A. Vilas-Zornoza, A. Jiménez-Velasco, J. I. Martin-Subero, L. Cordeu, L. Gárate, E. San José-Eneriz, G. Abizanda, P. Rodríguez-Otero, P. Fortes, J. Rifón, E. Bandrés, M. J. Calasanz, V. Martín, A. Heiniger, A. Torres, R. Siebert, J. Román-Gomez and F. Prósper (2009). "Epigenetic silencing of the tumor suppressor microRNA Hsa-miR-124a regulates CDK6 expression and confers a poor prognosis in acute lymphoblastic leukemia." Cancer Res 69(10): 4443-4453.
2. An, F., S. Yamanaka, S. Allen, L. R. Roberts, G. J. Gores, T. M. Pawlik, Q. Xie, M. Ishida, E. Mezey, A. C. Ferguson-Smith, Y. Mori and F. M. Selaru (2012). "Silencing of miR-370 in human cholangiocarcinoma by allelic loss and interleukin-6 induced maternal to paternal epigenotype switch." PLoS One 7(10): e45606.
3. Ando, T., T. Yoshida, S. Enomoto, K. Asada, M. Tatematsu, M. Ichinose, T. Sugiyama and T. Ushijima (2009). "DNA methylation of microRNA genes in gastric mucosae of gastric cancer patients: its possible involvement in the formation of epigenetic field defect." Int J Cancer 124(10): 2367-2374.
4. Andolfo, I., L. Liguori, P. De Antonellis, E. Cusanelli, F. Marinaro, F. Pistollato, L. Garzia, G. De Vita, G. Petrosino, B. Accordi, R. Migliorati, G. Basso, A. Iolascon, G. Cinalli and M. Zollo (2012). "The micro-RNA 199b-5p regulatory circuit involves Hes1, CD15, and epigenetic modifications in medulloblastoma." Neuro Oncol 14(5): 596-612.
5. Aprelikova, O., J. Palla, B. Hibler, X. Yu, Y. E. Greer, M. Yi, R. Stephens, G. L. Maxwell, A. Jazaeri, J. I. Risinger, J. S. Rubin and J. Niederhuber (2013). "Silencing of miR-148a in cancer-associated fibroblasts results in WNT10B-mediated stimulation of tumor cell motility." Oncogene 32(27): 3246-3253.
6. Asangani, I. A., P. W. Harms, L. Dodson, M. Pandhi, L. P. Kunju, C. A. Maher, D. R. Fullen, T. M. Johnson, T. J. Giordano, N. Palanisamy and A. M. Chinnaiyan (2012). "Genetic and epigenetic loss of microRNA-31 leads to feed-forward expression of EZH2 in melanoma." Oncotarget 3(9): 1011-1025.
7. Asuthkar, S., K. K. Velpula, C. Chetty, B. Gorantla and J. S. Rao (2012). "Epigenetic regulation of miRNA-211 by MMP-9 governs glioma cell apoptosis, chemosensitivity and radiosensitivity." Oncotarget 3(11): 1439-1454.
8. Augoff, K., B. McCue, E. F. Plow and K. Sossey-Alaoui (2012). "miR-31 and its host gene lncRNA LOC554202 are regulated by promoter hypermethylation in triple-negative breast cancer." Mol Cancer 11: 5.
9. Balaguer, F., A. Link, J. J. Lozano, M. Cuatrecasas, T. Nagasaka, C. R. Boland and A. Goel (2010). "Epigenetic silencing of miR-137 is an early event in colorectal carcinogenesis." Cancer Res 70(16): 6609-6618.
10. Bandres, E., X. Agirre, N. Bitarte, N. Ramirez, R. Zarate, J. Roman-Gomez, F. Prosper and J. Garcia-Foncillas (2009). "Epigenetic regulation of microRNA expression in colorectal cancer." Int J Cancer 125(11): 2737-2743.
11. Biagioni, F., N. Bossel Ben-Moshe, G. Fontemaggi, V. Canu, F. Mori, B. Antoniani, A. Di Benedetto, R. Santoro, S. Germoni, F. De Angelis, A. Cambria, R. Avraham, G. Grasso, S. Strano, P. Muti, M. Mottolese, Y. Yarden, E. Domany and G. Blandino (2012). "miR-10b*, a master inhibitor of the cell cycle, is down-regulated in human breast tumours." EMBO Mol Med 4(11): 1214-1229.
12. Bier, A., N. Giladi, N. Kronfeld, H. K. Lee, S. Cazacu, S. Finniss, C. Xiang, L. Poisson, A. C. deCarvalho, S. Slavin, E. Jacoby, M. Yalon, A. Toren, T. Mikkelsen and C. Brodie (2013). "MicroRNA-137 is downregulated in glioblastoma and inhibits the stemness of glioma stem cells by targeting RTVP-1." Oncotarget 4(5): 665-676.
13. Botezatu, A., C. D. Goia-Rusanu, I. V. Iancu, I. Huica, A. Plesa, D. Socolov, C. Ungureanu and G. Anton (2011). "Quantitative analysis of the relationship between microRNA‑124a, -34b and -203 gene methylation and cervical oncogenesis." Mol Med Report 4(1): 121-128.
14. Braconi, C., T. Kogure, N. Valeri, N. Huang, G. Nuovo, S. Costinean, M. Negrini, E. Miotto, C. M. Croce and T. Patel (2011). "microRNA-29 can regulate expression of the long non-coding RNA gene MEG3 in hepatocellular cancer." Oncogene 30(47): 4750-4756.
15. Brueckner, B., C. Stresemann, R. Kuner, C. Mund, T. Musch, M. Meister, H. Sültmann and F. Lyko (2007). "The human let-7a-3 locus contains an epigenetically regulated microRNA gene with oncogenic function." Cancer Res 67(4): 1419-1423.
16. Bueno, M. J., I. Pérez de Castro, M. Gómez de Cedrón, J. Santos, G. A. Calin, J. C. Cigudosa, C. M. Croce, J. Fernández-Piqueras and M. Malumbres (2008). "Genetic and epigenetic silencing of microRNA-203 enhances ABL1 and BCR-ABL1 oncogene expression." Cancer Cell 13(6): 496-506.
17. Cai, J., C. Yang, Q. Yang, H. Ding, J. Jia, J. Guo, J. Wang and Z. Wang (2013). "Deregulation of let-7e in epithelial ovarian cancer promotes the development of resistance to cisplatin." Oncogenesis 2: e75.
18. Cao, J., Y. Song, N. Bi, J. Shen, W. Liu, J. Fan, G. Sun, T. Tong, J. He, Y. Shi, X. Zhang, N. Lu, Y. He, H. Zhang, K. Ma, X. Luo, L. Lv, H. Deng, J. Cheng, J. Zhu, L. Wang and Q. Zhan (2013). "DNA methylation-mediated repression of miR-886-3p predicts poor outcome of human small cell lung cancer." Cancer Res 73(11): 3326-3335.
19. Castilla, M., J. Díaz-Martín, D. Sarrió, L. Romero-Pérez, M. López-García, B. Vieites, M. Biscuola, S. Ramiro-Fuentes, C. M. Isacke and J. Palacios (2012). "MicroRNA-200 family modulation in distinct breast cancer phenotypes." PLoS One 7(10): e47709.
20. Chen, Q., X. Chen, M. Zhang, Q. Fan, S. Luo and X. Cao (2011). "miR-137 is frequently down-regulated in gastric cancer and is a negative regulator of Cdc42." Dig Dis Sci 56(7): 2009-2016.
21. Chen, X., D. He, X. D. Dong, F. Dong, J. Wang, L. Wang, J. Tang, D. N. Hu, D. Yan and L. Tu (2013). "MicroRNA-124a is epigenetically regulated and acts as a tumor suppressor by controlling multiple targets in uveal melanoma." Invest Ophthalmol Vis Sci 54(3): 2248-2256.
22. Chen, X., H. Hu, X. Guan, G. Xiong, Y. Wang, K. Wang, J. Li, X. Xu, K. Yang and Y. Bai (2012). "CpG island methylation status of miRNAs in esophageal squamous cell carcinoma." Int J Cancer 130(7): 1607-1613.
23. Chen, X., J. Wang, H. Shen, J. Lu, C. Li, D. N. Hu, X. D. Dong, D. Yan and L. Tu (2011). "Epigenetics, microRNAs, and carcinogenesis: functional role of microRNA-137 in uveal melanoma." Invest Ophthalmol Vis Sci 52(3): 1193-1199.
24. Chen, X., L. Zhang, T. Zhang, M. Hao, X. Zhang, J. Zhang, Q. Xie, Y. Wang, M. Guo, H. Zhuang and F. Lu (2013). "Methylation-mediated repression of microRNA 129-2 enhances oncogenic SOX4 expression in HCC." Liver Int 33(3): 476-486.
25. Chim, C. S., T. S. Wan, K. Y. Wong, T. K. Fung, H. G. Drexler and K. F. Wong (2011). "Methylation of miR-34a, miR-34b/c, miR-124-1 and miR-203 in Ph-negative myeloproliferative neoplasms." J Transl Med 9: 197.
26. Chim, C. S., K. Y. Wong, C. Y. Leung, L. P. Chung, P. K. Hui, S. Y. Chan and L. Yu (2011). "Epigenetic inactivation of the hsa-miR-203 in haematological malignancies." J Cell Mol Med 15(12): 2760-2767.
27. Corney, D. C., C. I. Hwang, A. Matoso, M. Vogt, A. Flesken-Nikitin, A. K. Godwin, A. A. Kamat, A. K. Sood, L. H. Ellenson, H. Hermeking and A. Y. Nikitin (2010). "Frequent downregulation of miR-34 family in human ovarian cancers." Clin Cancer Res 16(4): 1119-1128.
28. Craig, V. J., S. B. Cogliatti, H. Rehrauer, T. Wündisch and A. Müller (2011). "Epigenetic silencing of microRNA-203 dysregulates ABL1 expression and drives Helicobacter-associated gastric lymphomagenesis." Cancer Res 71(10): 3616-3624.
29. Dang, J., Y. Q. Bian, J. Y. Sun, F. Chen, G. Y. Dong, Q. Liu, X. W. Wang, J. Kjems, S. Gao and Q. T. Wang (2013). "MicroRNA-137 promoter methylation in oral lichen planus and oral squamous cell carcinoma." J Oral Pathol Med 42(4): 315-321.
30. Dar, A. A., S. Majid, C. Rittsteuer, D. de Semir, V. Bezrookove, S. Tong, M. Nosrati, R. Sagebiel, J. R. Miller and M. Kashani-Sabet (2013). "The role of miR-18b in MDM2-p53 pathway signaling and melanoma progression." J Natl Cancer Inst 105(6): 433-442.
31. Datta, J., H. Kutay, M. W. Nasser, G. J. Nuovo, B. Wang, S. Majumder, C. G. Liu, S. Volinia, C. M. Croce, T. D. Schmittgen, K. Ghoshal and S. T. Jacob (2008). "Methylation mediated silencing of MicroRNA-1 gene and its role in hepatocellular carcinogenesis." Cancer Res 68(13): 5049-5058.
32. de Souza Rocha Simonini, P., A. Breiling, N. Gupta, M. Malekpour, M. Youns, R. Omranipour, F. Malekpour, S. Volinia, C. M. Croce, H. Najmabadi, S. Diederichs, O. Sahin, D. Mayer, F. Lyko, J. D. Hoheisel and Y. Riazalhosseini (2010). "Epigenetically deregulated microRNA-375 is involved in a positive feedback loop with estrogen receptor alpha in breast cancer cells." Cancer Res 70(22): 9175-9184.
33. Deng, H., Y. Guo, H. Song, B. Xiao, W. Sun, Z. Liu, X. Yu, T. Xia, L. Cui and J. Guo (2013). "MicroRNA-195 and microRNA-378 mediate tumor growth suppression by epigenetical regulation in gastric cancer." Gene 518(2): 351-359.
34. Doberstein, K., N. Steinmeyer, A. K. Hartmetz, W. Eberhardt, M. Mittelbronn, P. N. Harter, E. Juengel, R. Blaheta, J. Pfeilschifter and P. Gutwein (2013). "MicroRNA-145 targets the metalloprotease ADAM17 and is suppressed in renal cell carcinoma patients." Neoplasia 15(2): 218-230.
35. Dohi, O., K. Yasui, Y. Gen, H. Takada, M. Endo, K. Tsuji, C. Konishi, N. Yamada, H. Mitsuyoshi, N. Yagi, Y. Naito, S. Tanaka, S. Arii and T. Yoshikawa (2013). "Epigenetic silencing of miR-335 and its host gene MEST in hepatocellular carcinoma." Int J Oncol 42(2): 411-418.
36. Dong, F. and D. Lou (2012). "MicroRNA-34b/c suppresses uveal melanoma cell proliferation and migration through multiple targets." Mol Vis 18: 537-546.
37. Dudziec, E., S. Miah, H. M. Choudhry, H. C. Owen, S. Blizard, M. Glover, F. C. Hamdy and J. W. Catto (2011). "Hypermethylation of CpG islands and shores around specific microRNAs and mirtrons is associated with the phenotype and presence of bladder cancer." Clin Cancer Res 17(6): 1287-1296.
38. Eades, G., Y. Yao, M. Yang, Y. Zhang, S. Chumsri and Q. Zhou (2011). "miR-200a regulates SIRT1 expression and epithelial to mesenchymal transition (EMT)-like transformation in mammary epithelial cells." J Biol Chem 286(29): 25992-26002.
39. Endo, H., T. Muramatsu, M. Furuta, N. Uzawa, A. Pimkhaokham, T. Amagasa, J. Inazawa and K. Kozaki (2013). "Potential of tumor-suppressive miR-596 targeting LGALS3BP as a therapeutic agent in oral cancer." Carcinogenesis 34(3): 560-569.
40. Fazi, F., S. Racanicchi, G. Zardo, L. M. Starnes, M. Mancini, L. Travaglini, D. Diverio, E. Ammatuna, G. Cimino, F. Lo-Coco, F. Grignani and C. Nervi (2007). "Epigenetic silencing of the myelopoiesis regulator microRNA-223 by the AML1/ETO oncoprotein." Cancer Cell 12(5): 457-466.
41. Formosa, A., A. M. Lena, E. K. Markert, S. Cortelli, R. Miano, A. Mauriello, N. Croce, J. Vandesompele, P. Mestdagh, E. Finazzi-Agrò, A. J. Levine, G. Melino, S. Bernardini and E. Candi (2013). "DNA methylation silences miR-132 in prostate cancer." Oncogene 32(1): 127-134.
42. Furuta, M., K. I. Kozaki, S. Tanaka, S. Arii, I. Imoto and J. Inazawa (2010). "miR-124 and miR-203 are epigenetically silenced tumor-suppressive microRNAs in hepatocellular carcinoma." Carcinogenesis 31(5): 766-776.
43. Garzia, L., I. Andolfo, E. Cusanelli, N. Marino, G. Petrosino, D. De Martino, V. Esposito, A. Galeone, L. Navas, S. Esposito, S. Gargiulo, S. Fattet, V. Donofrio, G. Cinalli, A. Brunetti, L. D. Vecchio, P. A. Northcott, O. Delattre, M. D. Taylor, A. Iolascon and M. Zollo (2009). "MicroRNA-199b-5p impairs cancer stem cells through negative regulation of HES1 in medulloblastoma." PLoS One 4(3): e4998.
44. Gebauer, K., I. Peters, N. Dubrowinskaja, J. Hennenlotter, M. Abbas, R. Scherer, H. Tezval, A. S. Merseburger, A. Stenzl, M. A. Kuczyk and J. Serth (2013). "Hsa-mir-124-3 CpG island methylation is associated with advanced tumours and disease recurrence of patients with clear cell renal cell carcinoma." Br J Cancer 108(1): 131-138.
45. Geng, J., H. Luo, Y. Pu, Z. Zhou, X. Wu, W. Xu and Z. Yang (2012). "Methylation mediated silencing of miR-23b expression and its role in glioma stem cells." Neurosci Lett 528(2): 185-189.
46. Grady, W. M., R. K. Parkin, P. S. Mitchell, J. H. Lee, Y. H. Kim, K. D. Tsuchiya, M. K. Washington, C. Paraskeva, J. K. Willson, A. M. Kaz, E. M. Kroh, A. Allen, B. R. Fritz, S. D. Markowitz and M. Tewari (2008). "Epigenetic silencing of the intronic microRNA hsa-miR-342 and its host gene EVL in colorectal cancer." Oncogene 27(27): 3880-3888.
47. Guo, L. H., H. Li, F. Wang, J. Yu and J. S. He (2013). "The Tumor Suppressor Roles of miR-433 and miR-127 in Gastric Cancer." Int J Mol Sci 14(7): 14171-14184.
48. Hackanson, B., K. L. Bennett, R. M. Brena, J. Jiang, R. Claus, S. S. Chen, N. Blagitko-Dorfs, K. Maharry, S. P. Whitman, T. D. Schmittgen, M. Lübbert, G. Marcucci, C. D. Bloomfield and C. Plass (2008). "Epigenetic modification of CCAAT/enhancer binding protein alpha expression in acute myeloid leukemia." Cancer Res 68(9): 3142-3151.
49. Han, L., P. D. Witmer, E. Casey, D. Valle and S. Sukumar (2007). "DNA methylation regulates MicroRNA expression." Cancer Biol Ther 6(8): 1284-1288.
50. Hanoun, N., Y. Delpu, A. A. Suriawinata, B. Bournet, C. Bureau, J. Selves, G. J. Tsongalis, M. Dufresne, L. Buscail, P. Cordelier and J. Torrisani (2010). "The silencing of microRNA 148a production by DNA hypermethylation is an early event in pancreatic carcinogenesis." Clin Chem 56(7): 1107-1118.
51. Hashimoto, Y., Y. Akiyama, T. Otsubo, S. Shimada and Y. Yuasa (2010). "Involvement of epigenetically silenced microRNA-181c in gastric carcinogenesis." Carcinogenesis 31(5): 777-784.
52. He, Y., Y. Cui, W. Wang, J. Gu, S. Guo, K. Ma and X. Luo (2011). "Hypomethylation of the hsa-miR-191 locus causes high expression of hsa-mir-191 and promotes the epithelial-to-mesenchymal transition in hepatocellular carcinoma." Neoplasia 13(9): 841-853.
53. Hiroki, E., F. Suzuki, J. Akahira, S. Nagase, K. Ito, J. Sugawara, Y. Miki, T. Suzuki, H. Sasano and N. Yaegashi (2012). "MicroRNA-34b functions as a potential tumor suppressor in endometrial serous adenocarcinoma." Int J Cancer 131(4): E395-404.
54. Hu, H., S. Li, X. Cui, X. Lv, Y. Jiao, F. Yu, H. Yao, E. Song, Y. Chen, M. Wang and L. Lin (2013). "The overexpression of hypomethylated miR-663 induces chemotherapy resistance in human breast cancer cells by targeting heparin sulfate proteoglycan 2 (HSPG2)." J Biol Chem 288(16): 10973-10985.
55. Huang, Y. W., C. T. Kuo, J. H. Chen, P. J. Goodfellow, T. H. Huang, J. S. Rader and D. S. Uyar (2014). "Hypermethylation of miR-203 in endometrial carcinomas." Gynecol Oncol 133(2): 340-345.
56. Huang, Y. W., J. C. Liu, D. E. Deatherage, J. Luo, D. G. Mutch, P. J. Goodfellow, D. S. Miller and T. H. Huang (2009). "Epigenetic repression of microRNA-129-2 leads to overexpression of SOX4 oncogene in endometrial cancer." Cancer Res 69(23): 9038-9046.
57. Hulf, T., T. Sibbritt, E. D. Wiklund, S. Bert, D. Strbenac, A. L. Statham, M. D. Robinson and S. J. Clark (2011). "Discovery pipeline for epigenetically deregulated miRNAs in cancer: integration of primary miRNA transcription." BMC Genomics 12: 54.
58. Iorio, M. V., R. Visone, G. Di Leva, V. Donati, F. Petrocca, P. Casalini, C. Taccioli, S. Volinia, C. G. Liu, H. Alder, G. A. Calin, S. Ménard and C. M. Croce (2007). "MicroRNA signatures in human ovarian cancer." Cancer Res 67(18): 8699-8707.
59. Jung, C. J., S. Iyengar, K. R. Blahnik, T. P. Ajuha, J. X. Jiang, P. J. Farnham and M. Zern (2011). "Epigenetic modulation of miR-122 facilitates human embryonic stem cell self-renewal and hepatocellular carcinoma proliferation." PLoS One 6(11): e27740.
60. Kalimutho, M., S. Di Cecilia, G. Del Vecchio Blanco, F. Roviello, P. Sileri, M. Cretella, A. Formosa, G. Corso, D. Marrelli, F. Pallone, G. Federici and S. Bernardini (2011). "Epigenetically silenced miR-34b/c as a novel faecal-based screening marker for colorectal cancer." Br J Cancer 104(11): 1770-1778.
61. Kim, K., H. C. Lee, J. L. Park, M. Kim, S. Y. Kim, S. M. Noh, K. S. Song, J. C. Kim and Y. S. Kim (2011). "Epigenetic regulation of microRNA-10b and targeting of oncogenic MAPRE1 in gastric cancer." Epigenetics 6(6): 740-751.
62. Kim, S., U. J. Lee, M. N. Kim, E. J. Lee, J. Y. Kim, M. Y. Lee, S. Choung, Y. J. Kim and Y. C. Choi (2008). "MicroRNA miR-199a* regulates the MET proto-oncogene and the downstream extracellular signal-regulated kinase 2 (ERK2)." J Biol Chem 283(26): 18158-18166.
63. Kitano, K., K. Watanabe, N. Emoto, H. Kage, E. Hamano, T. Nagase, A. Sano, T. Murakawa, J. Nakajima, A. Goto, M. Fukayama, Y. Yatomi, N. Ohishi and D. Takai (2011). "CpG island methylation of microRNAs is associated with tumor size and recurrence of non-small-cell lung cancer." Cancer Sci 102(12): 2126-2131.
64. Kozaki, K., I. Imoto, S. Mogi, K. Omura and J. Inazawa (2008). "Exploration of tumor-suppressive microRNAs silenced by DNA hypermethylation in oral cancer." Cancer Res 68(7): 2094-2105.
65. Kubo, T., S. Toyooka, K. Tsukuda, M. Sakaguchi, T. Fukazawa, J. Soh, H. Asano, T. Ueno, T. Muraoka, H. Yamamoto, Y. Nasu, T. Kishimoto, H. I. Pass, H. Matsui, N. H. Huh and S. Miyoshi (2011). "Epigenetic silencing of microRNA-34b/c plays an important role in the pathogenesis of malignant pleural mesothelioma." Clin Cancer Res 17(15): 4965-4974.
66. Langevin, S. M., R. A. Stone, C. H. Bunker, J. R. Grandis, R. W. Sobol and E. Taioli (2010). "MicroRNA-137 promoter methylation in oral rinses from patients with squamous cell carcinoma of the head and neck is associated with gender and body mass index." Carcinogenesis 31(5): 864-870.
67. Lee, K. H., C. Lotterman, C. Karikari, N. Omura, G. Feldmann, N. Habbe, M. G. Goggins, J. T. Mendell and A. Maitra (2009). "Epigenetic silencing of MicroRNA miR-107 regulates cyclin-dependent kinase 6 expression in pancreatic cancer." Pancreatology 9(3): 293-301.
68. Lehmann, U., B. Hasemeier, M. Christgen, M. Müller, D. Römermann, F. Länger and H. Kreipe (2008). "Epigenetic inactivation of microRNA gene hsa-mir-9-1 in human breast cancer." J Pathol 214(1): 17-24.
69. Lei, H., D. Zou, Z. Li, M. Luo, L. Dong, B. Wang, H. Yin, Y. Ma, C. Liu, F. Wang, J. Zhang, J. Yu and Y. Li (2013). "MicroRNA-219-2-3p functions as a tumor suppressor in gastric cancer and is regulated by DNA methylation." PLoS One 8(4): e60369.
70. Li, C. L., H. Nie, M. Wang, L. P. Su, J. F. Li, Y. Y. Yu, M. Yan, Q. L. Qu, Z. G. Zhu and B. Y. Liu (2012). "microRNA-155 is downregulated in gastric cancer cells and involved in cell metastasis." Oncol Rep 27(6): 1960-1966.
71. Li, P., X. Chen, L. Su, C. Li, Q. Zhi, B. Yu, H. Sheng, J. Wang, R. Feng, Q. Cai, J. Li, Y. Yu, M. Yan, B. Liu and Z. Zhu (2013). "Epigenetic silencing of miR-338-3p contributes to tumorigenicity in gastric cancer by targeting SSX2IP." PLoS One 8(6): e66782.
72. Li, X., R. Lin and J. Li (2011). "Epigenetic silencing of microRNA-375 regulates PDK1 expression in esophageal cancer." Dig Dis Sci 56(10): 2849-2856.
73. Lin, P. C., Y. L. Chiu, S. Banerjee, K. Park, J. M. Mosquera, E. Giannopoulou, P. Alves, A. K. Tewari, M. B. Gerstein, H. Beltran, A. M. Melnick, O. Elemento, F. Demichelis and M. A. Rubin (2013). "Epigenetic repression of miR-31 disrupts androgen receptor homeostasis and contributes to prostate cancer progression." Cancer Res 73(3): 1232-1244.
74. Liu, L., K. Chen, J. Wu, L. Shi, B. Hu, S. Cheng, M. Li and L. Song (2013). "Downregulation of miR-452 promotes stem-like traits and tumorigenicity of gliomas." Clin Cancer Res 19(13): 3429-3438.
75. Liu, R. F., X. Xu, J. Huang, Q. L. Fei, F. Chen, Y. D. Li and Z. G. Han (2013). "Down-regulation of miR-517a and miR-517c promotes proliferation of hepatocellular carcinoma cells via targeting Pyk2." Cancer Lett 329(2): 164-173.
76. Liu, S., P. M. Howell and A. I. Riker (2012). "Up-Regulation of miR-182 Expression after Epigenetic Modulation of Human Melanoma Cells." Ann Surg Oncol.
77. Liu, Y., W. Zheng, Y. Song, W. Ma and H. Yin (2013). "Low expression of miR-196b enhances the expression of BCR-ABL1 and HOXA9 oncogenes in chronic myeloid leukemogenesis." PLoS One 8(7): e68442.
78. Lodygin, D., V. Tarasov, A. Epanchintsev, C. Berking, T. Knyazeva, H. Körner, P. Knyazev, J. Diebold and H. Hermeking (2008). "Inactivation of miR-34a by aberrant CpG methylation in multiple types of cancer." Cell Cycle 7(16): 2591-2600.
79. Lu, C. Y., K. Y. Lin, M. T. Tien, C. T. Wu, Y. H. Uen and T. L. Tseng (2013). "Frequent DNA methylation of MiR-129-2 and its potential clinical implication in hepatocellular carcinoma." Genes Chromosomes Cancer 52(7): 636-643.
80. Lu, L., D. Katsaros, I. A. de la Longrais, O. Sochirca and H. Yu (2007). "Hypermethylation of let-7a-3 in epithelial ovarian cancer is associated with low insulin-like growth factor-II expression and favorable prognosis." Cancer Res 67(21): 10117-10122.
81. Lu, L., D. Katsaros, Y. Zhu, A. Hoffman, S. Luca, C. E. Marion, L. Mu, H. Risch and H. Yu (2011). "Let-7a regulation of insulin-like growth factors in breast cancer." Breast Cancer Res Treat 126(3): 687-694.
82. Lujambio, A., S. Ropero, E. Ballestar, M. F. Fraga, C. Cerrato, F. Setién, S. Casado, A. Suarez-Gauthier, M. Sanchez-Cespedes, A. Git, A. Gitt, I. Spiteri, P. P. Das, C. Caldas, E. Miska and M. Esteller (2007). "Genetic unmasking of an epigenetically silenced microRNA in human cancer cells." Cancer Res 67(4): 1424-1429.
83. Majid, S., A. A. Dar, S. Saini, S. Arora, V. Shahryari, M. S. Zaman, I. Chang, S. Yamamura, Y. Tanaka, G. Deng and R. Dahiya (2012). "miR-23b represses proto-oncogene Src kinase and functions as methylation-silenced tumor suppressor with diagnostic and prognostic significance in prostate cancer." Cancer Res 72(24): 6435-6446.
84. Majid, S., A. A. Dar, S. Saini, V. Shahryari, S. Arora, M. S. Zaman, I. Chang, S. Yamamura, Y. Tanaka, T. Chiyomaru, G. Deng and R. Dahiya (2013). "miRNA-34b inhibits prostate cancer through demethylation, active chromatin modifications, and AKT pathways." Clin Cancer Res 19(1): 73-84.
85. Mazar, J., D. DeBlasio, S. S. Govindarajan, S. Zhang and R. J. Perera (2011). "Epigenetic regulation of microRNA-375 and its role in melanoma development in humans." FEBS Lett 585(15): 2467-2476.
86. Mazar, J., D. Khaitan, D. DeBlasio, C. Zhong, S. S. Govindarajan, S. Kopanathi, S. Zhang, A. Ray and R. J. Perera (2011). "Epigenetic regulation of microRNA genes and the role of miR-34b in cell invasion and motility in human melanoma." PLoS One 6(9): e24922.
87. Meng, F., H. Wehbe-Janek, R. Henson, H. Smith and T. Patel (2008). "Epigenetic regulation of microRNA-370 by interleukin-6 in malignant human cholangiocytes." Oncogene 27(3): 378-386.
88. Minor, J., X. Wang, F. Zhang, J. Song, A. Jimeno, X. J. Wang, X. Lu, N. Gross, M. Kulesz-Martin, D. Wang and S. L. Lu (2012). "Methylation of microRNA-9 is a specific and sensitive biomarker for oral and oropharyngeal squamous cell carcinomas." Oral Oncol 48(1): 73-78.
89. Misiewicz-Krzeminska, I., M. E. Sarasquete, D. Quwaider, P. Krzeminski, F. V. Ticona, T. Paíno, M. Delgado, A. Aires, E. M. Ocio, R. García-Sanz, J. F. San Miguel and N. C. Gutiérrez (2013). "Restoration of microRNA-214 expression reduces growth of myeloma cells through positive regulation of P53 and inhibition of DNA replication." Haematologica 98(4): 640-648.
90. Omura, N., C. P. Li, A. Li, S. M. Hong, K. Walter, A. Jimeno, M. Hidalgo and M. Goggins (2008). "Genome-wide profiling of methylated promoters in pancreatic adenocarcinoma." Cancer Biol Ther 7(7): 1146-1156.
91. Pallasch, C. P., M. Patz, Y. J. Park, S. Hagist, D. Eggle, R. Claus, S. Debey-Pascher, A. Schulz, L. P. Frenzel, J. Claasen, N. Kutsch, G. Krause, C. Mayr, A. Rosenwald, C. Plass, J. L. Schultze, M. Hallek and C. M. Wendtner (2009). "miRNA deregulation by epigenetic silencing disrupts suppression of the oncogene PLAG1 in chronic lymphocytic leukemia." Blood 114(15): 3255-3264.
92. Pichiorri, F., S. S. Suh, A. Rocci, L. De Luca, C. Taccioli, R. Santhanam, W. Zhou, D. M. Benson, C. Hofmainster, H. Alder, M. Garofalo, G. Di Leva, S. Volinia, H. J. Lin, D. Perrotti, M. Kuehl, R. I. Aqeilan, A. Palumbo and C. M. Croce (2010). "Downregulation of p53-inducible microRNAs 192, 194, and 215 impairs the p53/MDM2 autoregulatory loop in multiple myeloma development." Cancer Cell 18(4): 367-381.
93. Pigazzi, M., E. Manara, E. Baron and G. Basso (2009). "miR-34b targets cyclic AMP-responsive element binding protein in acute myeloid leukemia." Cancer Res 69(6): 2471-2478.
94. Pigazzi, M., E. Manara, S. Bresolin, C. Tregnago, A. Beghin, E. Baron, E. Giarin, E. C. Cho, R. Masetti, D. S. Rao, K. M. Sakamoto and G. Basso (2013). "MicroRNA-34b promoter hypermethylation induces CREB overexpression and contributes to myeloid transformation." Haematologica 98(4): 602-610.
95. Png, K. J., M. Yoshida, X. H. Zhang, W. Shu, H. Lee, A. Rimner, T. A. Chan, E. Comen, V. P. Andrade, S. W. Kim, T. A. King, C. A. Hudis, L. Norton, J. Hicks, J. Massagué and S. F. Tavazoie (2011). "MicroRNA-335 inhibits tumor reinitiation and is silenced through genetic and epigenetic mechanisms in human breast cancer." Genes Dev 25(3): 226-231.
96. Ragusa, M., A. Majorana, B. Banelli, D. Barbagallo, L. Statello, I. Casciano, M. R. Guglielmino, L. R. Duro, M. Scalia, G. Magro, C. Di Pietro, M. Romani and M. Purrello (2010). "MIR152, MIR200B, and MIR338, human positional and functional neuroblastoma candidates, are involved in neuroblast differentiation and apoptosis." J Mol Med (Berl) 88(10): 1041-1053.
97. Rauhala, H. E., S. E. Jalava, J. Isotalo, H. Bracken, S. Lehmusvaara, T. L. Tammela, H. Oja and T. Visakorpi (2010). "miR-193b is an epigenetically regulated putative tumor suppressor in prostate cancer." Int J Cancer 127(6): 1363-1372.
98. Roman-Gomez, J., X. Agirre, A. Jiménez-Velasco, V. Arqueros, A. Vilas-Zornoza, P. Rodriguez-Otero, I. Martin-Subero, L. Garate, L. Cordeu, E. San José-Eneriz, V. Martin, J. A. Castillejo, E. Bandrés, M. J. Calasanz, R. Siebert, A. Heiniger, A. Torres and F. Prosper (2009). "Epigenetic regulation of microRNAs in acute lymphoblastic leukemia." J Clin Oncol 27(8): 1316-1322.
99. Roy, S., E. Levi, A. P. Majumdar and F. H. Sarkar (2012). "Expression of miR-34 is lost in colon cancer which can be re-expressed by a novel agent CDF." J Hematol Oncol 5: 58.
100. Saito, Y., G. Liang, G. Egger, J. M. Friedman, J. C. Chuang, G. A. Coetzee and P. A. Jones (2006). "Specific activation of microRNA-127 with downregulation of the proto-oncogene BCL6 by chromatin-modifying drugs in human cancer cells." Cancer Cell 9(6): 435-443.
101. Saito, Y., H. Suzuki, H. Tsugawa, I. Nakagawa, J. Matsuzaki, Y. Kanai and T. Hibi (2009). "Chromatin remodeling at Alu repeats by epigenetic treatment activates silenced microRNA-512-5p with downregulation of Mcl-1 in human gastric cancer cells." Oncogene 28(30): 2738-2744.
102. Shen, R., S. Pan, S. Qi, X. Lin and S. Cheng (2010). "Epigenetic repression of microRNA-129-2 leads to overexpression of SOX4 in gastric cancer." Biochem Biophys Res Commun 394(4): 1047-1052.
103. Shimizu, T., H. Suzuki, M. Nojima, H. Kitamura, E. Yamamoto, R. Maruyama, M. Ashida, T. Hatahira, M. Kai, N. Masumori, T. Tokino, K. Imai, T. Tsukamoto and M. Toyota (2013). "Methylation of a panel of microRNA genes is a novel biomarker for detection of bladder cancer." Eur Urol 63(6): 1091-1100.
104. Shin, K. H., S. D. Bae, H. S. Hong, R. H. Kim, M. K. Kang and N. H. Park (2011). "miR-181a shows tumor suppressive effect against oral squamous cell carcinoma cells by downregulating K-ras." Biochem Biophys Res Commun 404(4): 896-902.
105. Siemens, H., J. Neumann, R. Jackstadt, U. Mansmann, D. Horst, T. Kirchner and H. Hermeking (2013). "Detection of miR-34a promoter methylation in combination with elevated expression of c-Met and β-catenin predicts distant metastasis of colon cancer." Clin Cancer Res 19(3): 710-720.
106. Silber, J., D. A. Lim, C. Petritsch, A. I. Persson, A. K. Maunakea, M. Yu, S. R. Vandenberg, D. G. Ginzinger, C. D. James, J. F. Costello, G. Bergers, W. A. Weiss, A. Alvarez-Buylla and J. G. Hodgson (2008). "miR-124 and miR-137 inhibit proliferation of glioblastoma multiforme cells and induce differentiation of brain tumor stem cells." BMC Med 6: 14.
107. Soto-Reyes, E., R. González-Barrios, F. Cisneros-Soberanis, R. Herrera-Goepfert, V. Pérez, D. Cantú, D. Prada, C. Castro, F. Recillas-Targa and L. A. Herrera (2012). "Disruption of CTCF at the miR-125b1 locus in gynecological cancers." BMC Cancer 12: 40.
108. Sun, J., Y. Song, Z. Wang, G. Wang, P. Gao, X. Chen, Z. Gao and H. Xu (2014). "Clinical significance of promoter region hypermethylation of microRNA-148a in gastrointestinal cancers." Onco Targets Ther 7: 853-863.
109. Sun, Q., J. Zhang, W. Cao, X. Wang, Q. Xu, M. Yan, X. Wu and W. Chen (2013). "Dysregulated miR-363 affects head and neck cancer invasion and metastasis by targeting podoplanin." Int J Biochem Cell Biol 45(3): 513-520.
110. Suzuki, H., E. Yamamoto, M. Nojima, M. Kai, H. O. Yamano, K. Yoshikawa, T. Kimura, T. Kudo, E. Harada, T. Sugai, H. Takamaru, T. Niinuma, R. Maruyama, H. Yamamoto, T. Tokino, K. Imai, M. Toyota and Y. Shinomura (2010). "Methylation-associated silencing of microRNA-34b/c in gastric cancer and its involvement in an epigenetic field defect." Carcinogenesis 31(12): 2066-2073.
111. Szenthe, K., A. Koroknai, F. Banati, Z. Bathori, R. Lozsa, J. Burgyan, H. Wolf, D. Salamon, K. Nagy, H. H. Niller and J. Minarovits (2013). "The 5' regulatory sequences of active miR-146a promoters are hypomethylated and associated with euchromatic histone modification marks in B lymphoid cells." Biochem Biophys Res Commun 433(4): 489-495.
112. Takahashi, M., M. Cuatrecasas, F. Balaguer, K. Hur, Y. Toiyama, A. Castells, C. R. Boland and A. Goel (2012). "The Clinical Significance of MiR-148a as a Predictive Biomarker in Patients with Advanced Colorectal Cancer." PLoS One 7(10): e46684.
113. Tanaka, N., S. Toyooka, J. Soh, T. Kubo, H. Yamamoto, Y. Maki, T. Muraoka, K. Shien, M. Furukawa, T. Ueno, H. Asano, K. Tsukuda, K. Aoe and S. Miyoshi (2012). "Frequent methylation and oncogenic role of microRNA-34b/c in small-cell lung cancer." Lung Cancer 76(1): 32-38.
114. Tanaka, T., M. Arai, S. Wu, T. Kanda, H. Miyauchi, F. Imazeki, H. Matsubara and O. Yokosuka (2011). "Epigenetic silencing of microRNA-373 plays an important role in regulating cell proliferation in colon cancer." Oncol Rep 26(5): 1329-1335.
115. Tang, J. T., J. L. Wang, W. Du, J. Hong, S. L. Zhao, Y. C. Wang, H. Xiong, H. M. Chen and J. Y. Fang (2011). "MicroRNA 345, a methylation-sensitive microRNA is involved in cell proliferation and invasion in human colorectal cancer." Carcinogenesis 32(8): 1207-1215.
116. Toyota, M., H. Suzuki, Y. Sasaki, R. Maruyama, K. Imai, Y. Shinomura and T. Tokino (2008). "Epigenetic silencing of microRNA-34b/c and B-cell translocation gene 4 is associated with CpG island methylation in colorectal cancer." Cancer Res 68(11): 4123-4132.
117. Tsai, K. W., H. W. Kao, H. C. Chen, S. J. Chen and W. C. Lin (2009). "Epigenetic control of the expression of a primate-specific microRNA cluster in human cancer cells." Epigenetics 4(8): 587-592.
118. Tsai, K. W., Y. L. Liao, C. W. Wu, L. Y. Hu, S. C. Li, W. C. Chan, M. R. Ho, C. H. Lai, H. W. Kao, W. L. Fang, K. H. Huang and W. C. Lin (2011). "Aberrant hypermethylation of miR-9 genes in gastric cancer." Epigenetics 6(10): 1189-1197.
119. Tsai, K. W., C. W. Wu, L. Y. Hu, S. C. Li, Y. L. Liao, C. H. Lai, H. W. Kao, W. L. Fang, K. H. Huang, W. C. Chan and W. C. Lin (2011). "Epigenetic regulation of miR-34b and miR-129 expression in gastric cancer." Int J Cancer 129(11): 2600-2610.
120. Tsuruta, T., K. Kozaki, A. Uesugi, M. Furuta, A. Hirasawa, I. Imoto, N. Susumu, D. Aoki and J. Inazawa (2011). "miR-152 is a tumor suppressor microRNA that is silenced by DNA hypermethylation in endometrial cancer." Cancer Res 71(20): 6450-6462.
121. Van Beers, B., J. Pringot, J. F. Gigot, J. Dautrebande and P. Mathurin (1990). "Nontumorous attenuation differences on computed tomographic portography." Gastrointest Radiol 15(2): 107-111.
122. Vogt, M., J. Munding, M. Grüner, S. T. Liffers, B. Verdoodt, J. Hauk, L. Steinstraesser, A. Tannapfel and H. Hermeking (2011). "Frequent concomitant inactivation of miR-34a and miR-34b/c by CpG methylation in colorectal, pancreatic, mammary, ovarian, urothelial, and renal cell carcinomas and soft tissue sarcomas." Virchows Arch 458(3): 313-322.
123. Vrba, L., T. J. Jensen, J. C. Garbe, R. L. Heimark, A. E. Cress, S. Dickinson, M. R. Stampfer and B. W. Futscher (2010). "Role for DNA methylation in the regulation of miR-200c and miR-141 expression in normal and cancer cells." PLoS One 5(1): e8697.
124. Vrba, L., J. L. Muñoz-Rodríguez, M. R. Stampfer and B. W. Futscher (2013). "miRNA gene promoters are frequent targets of aberrant DNA methylation in human breast cancer." PLoS One 8(1): e54398.
125. Wang, J., B. Yang, L. Han, X. Li, H. Tao, S. Zhang and Y. Hu (2013). "Demethylation of miR-9-3 and miR-193a genes suppresses proliferation and promotes apoptosis in non-small cell lung cancer cell lines." Cell Physiol Biochem 32(6): 1707-1719.
126. Wang, L. Q., Y. L. Kwong, C. S. Kho, K. F. Wong, K. Y. Wong, M. Ferracin, G. A. Calin and C. S. Chim (2013). "Epigenetic inactivation of miR-9 family microRNAs in chronic lymphocytic leukemia--implications on constitutive activation of NFκB pathway." Mol Cancer 12: 173.
127. Watanabe, K., N. Emoto, E. Hamano, M. Sunohara, M. Kawakami, H. Kage, K. Kitano, J. Nakajima, A. Goto, M. Fukayama, T. Nagase, Y. Yatomi, N. Ohishi and D. Takai (2012). "Genome structure-based screening identified epigenetically silenced microRNA associated with invasiveness in non-small-cell lung cancer." Int J Cancer 130(11): 2580-2590.
128. Weber, B., C. Stresemann, B. Brueckner and F. Lyko (2007). "Methylation of human microRNA genes in normal and neoplastic cells." Cell Cycle 6(9): 1001-1005.
129. Wiklund, E. D., S. Gao, T. Hulf, T. Sibbritt, S. Nair, D. E. Costea, S. B. Villadsen, V. Bakholdt, J. B. Bramsen, J. A. Sørensen, A. Krogdahl, S. J. Clark and J. Kjems (2011). "MicroRNA alterations and associated aberrant DNA methylation patterns across multiple sample types in oral squamous cell carcinoma." PLoS One 6(11): e27840.
130. Wiklund, E. D., J. Kjems and S. J. Clark (2010). "Epigenetic architecture and miRNA: reciprocal regulators." Epigenomics 2(6): 823-840.
131. Wilting, S. M., R. A. van Boerdonk, F. E. Henken, C. J. Meijer, B. Diosdado, G. A. Meijer, C. le Sage, R. Agami, P. J. Snijders and R. D. Steenbergen (2010). "Methylation-mediated silencing and tumour suppressive function of hsa-miR-124 in cervical cancer." Mol Cancer 9: 167.
132. Wong, K. Y., R. Liang, C. C. So, D. Y. Jin, J. F. Costello and C. S. Chim (2011). "Epigenetic silencing of MIR203 in multiple myeloma." Br J Haematol 154(5): 569-578.
133. Wong, K. Y., C. C. So, F. Loong, L. P. Chung, W. W. Lam, R. Liang, G. K. Li, D. Y. Jin and C. S. Chim (2011). "Epigenetic inactivation of the miR-124-1 in haematological malignancies." PLoS One 6(4): e19027.
134. Wong, K. Y., R. L. Yim, Y. L. Kwong, C. Y. Leung, P. K. Hui, F. Cheung, R. Liang, D. Y. Jin and C. S. Chim (2013). "Epigenetic inactivation of the MIR129-2 in hematological malignancies." J Hematol Oncol 6: 16.
135. Wong, K. Y., R. L. Yim, C. C. So, D. Y. Jin, R. Liang and C. S. Chim (2011). "Epigenetic inactivation of the MIR34B/C in multiple myeloma." Blood 118(22): 5901-5904.
136. Xu, Q., Y. Jiang, Y. Yin, Q. Li, J. He, Y. Jing, Y. T. Qi, W. Li, B. Lu, S. S. Peiper, B. H. Jiang and L. Z. Liu (2013). "A regulatory circuit of miR-148a/152 and DNMT1 in modulating cell transformation and tumor angiogenesis through IGF-IR and IRS1." J Mol Cell Biol 5(1): 3-13.
137. Yan, H., A. J. Choi, B. H. Lee and A. H. Ting (2011). "Identification and functional analysis of epigenetically silenced microRNAs in colorectal cancer cells." PLoS One 6(6): e20628.
138. Yan-Fang, T., N. Jian, L. Jun, W. Na, X. Pei-Fang, Z. Wen-Li, W. Dong, P. Li, W. Jian, F. Xing and P. Jian (2013). "The promoter of miR-663 is hypermethylated in Chinese pediatric acute myeloid leukemia (AML)." BMC Med Genet 14: 74.
139. Yang, C., J. Cai, Q. Wang, H. Tang, J. Cao, L. Wu and Z. Wang (2012). "Epigenetic silencing of miR-130b in ovarian cancer promotes the development of multidrug resistance by targeting colony-stimulating factor 1." Gynecol Oncol 124(2): 325-334.
140. Yao, T., Q. Rao, L. Liu, C. Zheng, Q. Xie, J. Liang and Z. Lin (2013). "Exploration of tumor-suppressive microRNAs silenced by DNA hypermethylation in cervical cancer." Virol J 10: 175.
141. Ying, Z., Y. Li, J. Wu, X. Zhu, Y. Yang, H. Tian, W. Li, B. Hu, S. Y. Cheng and M. Li (2013). "Loss of miR-204 expression enhances glioma migration and stem cell-like phenotype." Cancer Res 73(2): 990-999.
142. Yoshitomi, T., K. Kawakami, H. Enokida, T. Chiyomaru, I. Kagara, S. Tatarano, H. Yoshino, H. Arimura, K. Nishiyama, N. Seki and M. Nakagawa (2011). "Restoration of miR-517a expression induces cell apoptosis in bladder cancer cell lines." Oncol Rep 25(6): 1661-1668.
143. Yu, F., Y. Jiao, Y. Zhu, Y. Wang, J. Zhu, X. Cui, Y. Liu, Y. He, E. Y. Park, H. Zhang, X. Lv, K. Ma, F. Su, J. H. Park and E. Song (2012). "MicroRNA 34c gene down-regulation via DNA methylation promotes self-renewal and epithelial-mesenchymal transition in breast tumor-initiating cells." J Biol Chem 287(1): 465-473.
144. Zaman, M. S., Y. Chen, G. Deng, V. Shahryari, S. O. Suh, S. Saini, S. Majid, J. Liu, G. Khatri, Y. Tanaka and R. Dahiya (2010). "The functional significance of microRNA-145 in prostate cancer." Br J Cancer 103(2): 256-264.
145. Zehavi, L., R. Avraham, A. Barzilai, D. Bar-Ilan, R. Navon, Y. Sidi, D. Avni and R. Leibowitz-Amit (2012). "Silencing of a large microRNA cluster on human chromosome 14q32 in melanoma: biological effects of mir-376a and mir-376c on insulin growth factor 1 receptor." Mol Cancer 11: 44.
146. Zhang, L., S. Volinia, T. Bonome, G. A. Calin, J. Greshock, N. Yang, C. G. Liu, A. Giannakakis, P. Alexiou, K. Hasegawa, C. N. Johnstone, M. S. Megraw, S. Adams, H. Lassus, J. Huang, S. Kaur, S. Liang, P. Sethupathy, A. Leminen, V. A. Simossis, R. Sandaltzopoulos, Y. Naomoto, D. Katsaros, P. A. Gimotty, A. DeMichele, Q. Huang, R. Bützow, A. K. Rustgi, B. L. Weber, M. J. Birrer, A. G. Hatzigeorgiou, C. M. Croce and G. Coukos (2008). "Genomic and epigenetic alterations deregulate microRNA expression in human epithelial ovarian cancer." Proc Natl Acad Sci U S A 105(19): 7004-7009.
147. Zhang, Y., L. X. Yan, Q. N. Wu, Z. M. Du, J. Chen, D. Z. Liao, M. Y. Huang, J. H. Hou, Q. L. Wu, M. S. Zeng, W. L. Huang, Y. X. Zeng and J. Y. Shao (2011). "miR-125b is methylated and functions as a tumor suppressor by regulating the ETS1 proto-oncogene in human invasive breast cancer." Cancer Res 71(10): 3552-3562.
148. Zhang, Z., B. Zhang, W. Li, L. Fu, Z. Zhu and J. T. Dong (2011). "Epigenetic Silencing of miR-203 Upregulates SNAI2 and Contributes to the Invasiveness of Malignant Breast Cancer Cells." Genes Cancer 2(8): 782-791.
149. Zhu, A., J. Xia, J. Zuo, S. Jin, H. Zhou, L. Yao, H. Huang and Z. Han (2012). "MicroRNA-148a is silenced by hypermethylation and interacts with DNA methyltransferase 1 in gastric cancer." Med Oncol 29(4): 2701-2709.
150. Zhu, X., Y. Li, H. Shen, H. Li, L. Long, L. Hui and W. Xu (2013). "miR-137 inhibits the proliferation of lung cancer cells by targeting Cdc42 and Cdk6." FEBS Lett 587(1): 73-81.

© 2015 by the authors; licensee MDPI, Basel, Switzerland. This article is an open access article distributed under the terms and conditions of the Creative Commons Attribution license (http://creativecommons.org/licenses/by/4.0/).
